# The impact of social inequalities on children’s knowledge and representation of health and cancer

**DOI:** 10.1007/s00431-018-3173-3

**Published:** 2018-05-28

**Authors:** Véronique Régnier Denois, Aurelie Bourmaud, Mabrouk Nekaa, Céline Bezzaz, Véronique Bousser, Julie Kalecinski, Julia Dumesnil, Fabien Tinquaut, Dominique Berger, Franck Chauvin

**Affiliations:** 1 Hygée Center, Lucien Neuwirth Cancer Institute, Inserm, CIC1408, 108bis Av Albert Raimond, 42271 St Priest en Jarez, France; 2 Univ. Lyon, HESPER EA 7425, 42023 Saint-Etienne, France; 3 National Education, Lyon regional education authority, 92 rue de Marseille, 69007 Lyon, France

**Keywords:** School health, Developmental/behavioral issues, Cancer, Prevention, Health inequalities

## Abstract

Reducing inequalities in the field of cancer involves studying the knowledge and mental representations of cancer among children. A qualitative study was conducted on 191 children aged 9 to 12 using the “write and draw” technique to get spontaneous mental representations of “healthy things”, “unhealthy things” and “cancer”. We grouped the voluntary schools according to two deprivation levels. In response to the request to “write or draw anything you think keeps you healthy”, the main responses categories were physical activity, healthy food and basic needs. Smoking, drinking alcohol, sedentary lifestyles/lack of sport were identified as “unhealthy”. The first theme associated with “cancer” is the “cancer site” implying children have a segmented perception of cancer. Deprived children have radically different views about the key items representing cancer: they are more likely to believe the illness is systematically deadly. They are less likely to believe it is a treatable illness. They are less likely to associate cancer with risky behaviors, particularly alcohol consumption.

*Conclusion*: Social inequalities affect representations of cancer and health literacy from early childhood. Prevention programs taking into account these representations need to be introduced at school.

**Table:** 

**What is Known:** *• Social inequalities for cancer mortality are observed in all European countries and are particularly pronounced in France.* *• Reducing these inequalities in prevention programs implies studying the knowledge and mental representations of cancer among children.*	
**What is New:** *• This study identified representations of cancer in young children according to social level.* *• At age 9, children living in deprived areas are less able to produce content in discussions about cancer and have narrower mental representations and a more fatalistic view.*

## Introduction

France has a longer life expectancy than other European countries, but with significant inequalities: between 2000 and 2008, 35-year-old men in senior management roles had a life expectancy of 49 years, which is 6 years more than blue-collar workers [30]. Indeed, the mortality rate inequality index has risen from 1.5 to 2.5 in the last 20 years, comparing the lowest and highest levels of qualification [15]. This is the result of social inequalities in living conditions starting in childhood and concern education, jobs, housing, and social relationships, among others.

### Health literacy and social inequalities

Another factor affecting behaviors and beliefs about health is health literacy [26, 27, 31]. Health literacy is embedded within the social, cultural, and environmental context and norms [25]. Poor health literacy has been associated with health inequalities [12]. The only one study assessing the impact of health literacy on children concluded that health literacy enhancement had a major positive effect on health outcomes for this specific population [24]. Elements of literacy can be found in representations. Representations are defined as “a system of values, ideas and practices having for primary function to establish an order which will enable individuals to orient themselves in their material and social world and to master it, and for secondary function to enable communication to take place among the members of a community by providing them with a collective elaboration of a social object” [7]. Studying representations allows us to anticipate the social distribution of the elements of literacy, to understand why some elements are selected whereas others are rejected and how the different elements make sense in a coherent system.

### Health representations

Few studies about children’s health representations have been performed. The few studies existing focused mainly on children with a disease. The rare studies conducted on the general population mainly focused on in addiction among children and overweight [23]. Among those studies, the health representations identified were considered to be rich, and children mainly viewed health as a personal responsibility attributed to will-power. Yet, those representations were rarely connected to child health-related behaviors.

### Cancer representations

Studies of cancer representations have chiefly focused on adult populations. They concluded that cancer is considered a “distinct” disease for which the diagnosis is always synonymous with death in the collective psyche and “hides solid enduring beliefs that can produce dangerous behaviors for health” [18]. Children of that age already appear to have representations of cancer [4]. But most of the representations identified were focused on risk factors [2, 28].Other dimensions of children’s representations remain unexplored: illness stigma, attribution, disclosure.

Reducing inequalities in the field of cancer prevention implies studying the knowledge and the representations of cancer among children. [10, 29]. The representations are activated when the child comes into contact with illness or with information on cancer. These representations create the core understanding that will influence the child’s attitudes and current/future decisions in reacting to prevention stimuli, such as a smoking cessation campaign or screening incentive. This study therefore has the dual aim of identifying representations of health and cancer among 9 to 12-year-old children in a school setting, and determining whether the prevalence of some cancer representations is associated with the schools’ deprivation status.

## Methods

A qualitative study was conducted on children aged 9 to 12 in elementary and junior high schools. In order to help the children understand the research questions and communicate elements of their representations [21] even when the theme relates to a subject they might find difficult to discuss, we adopted a relatively non-threatening means of eliciting ideas: the “write and draw” technique, as used by A. Oakley in 1995 [17] in his research into cancer representations among children. The write and draw method was also used in other studies concerning children’s representations of similar subjects, as this enables the rapid production of large volumes of spontaneous, qualitative data. In addition, it avoids imposing overly academic means of self-expression [16]. Data collection was followed by a group discussion, the main aim of which being to relieve any anxiety among the children. The school nurses were able to spot children that may need specific support and this was organized in conjunction with their class teacher.

### Sample

The data was collected from a sample of elementary and junior high school children aged 9–12. All children from the targeted school classes (corresponding to age 9–12) of included schools were asked to participate. The schools were selected as follows: the study protocol was presented at an annual county meeting for school nurses (Loire department). The project was then proposed to all attending school nurses, independently of the geographic location of their school and therefore independently of its deprivation status. Volunteer school nurses had to obtain the school headmaster’s agreement. The schools included in the study received the intervention consecutively, the latter being blind to their deprivation status. The schools and, by then, the children included in the study were then ranked according to the social deprivation level of their geographic location. A deprivation index was calculated for each school, using the French adaptation of European Deprivation Index (EDI). The EDI is an adaptable transnational ecological deprivation index. It has been developed according to a common definition of deprivation—physical and social—while maintaining the specificity of each country. This index combines individual data from a European survey on poverty launched by the European Commission (EU-SILC) and data from the population census of each country. Those characteristics allow this index to be transposable from one country to another. This ecological deprivation index was developed for France by Pornet et al. [20] and includes 11 variables such as educational level, access to heating, access to a car, nationality, occupational category, number of persons in household, tenure and employment status. This index ranks individuals by residential areas based on five quintiles: ranging from most deprived (5) to least deprived (1). In this report, we grouped the schools according to two deprivation levels, with one “deprived” group, covering quintiles four and five and a “non-deprived” group, covering quintiles three down to 1.

### Data collection

The researcher was given access to at least one class in each school for a 90-min interview. The researchers addressed half classes each time. Children were asked to answer three questions using the write or draw method: “write or draw anything you think makes you healthy;” “write or draw anything you think makes you unhealthy;” and “write or draw anything you know about cancer.” They were informed that there were no “right or wrong” answers, and that they could answer any way they liked, including handing in a blank sheet with no answers.

Additional individual demographic data (age, sex, school year) were collected at the end of the interview.

### Analysis

A qualitative-quantitative mixed method was chosen to perform the analyses.

Qualitative blind assessments of the children’s creations were conducted by two independent readers. In the event of discrepancies between the two interpretations, a third reader was asked to settle the issue of classification.

The creations associated with good/bad health, were classified using the themes developed by Oakley et al. [16]. The sub theme “good health” was subdivided into the following themes: healthy food, exercise and sports, hygiene, not smoking, and sleeping. The sub theme “bad health” was subdivided into the following themes: diet, environment, violence, hygiene, alcohol, and medication. New themes emanated from this analysis were not found in the Oakley study. They have been added to the Oakley themes in the present analysis.

The third set of creations, concerning cancer, was analyzed using grounded theory approach [5]. This method relies on the construction of theory through the methodological gathering and analysis of data. Here, the focus was on the children’s creations: each researcher reviewed the creations collected and identified repeated concepts that became apparent. Those concepts were elements of meaning representing an idea. The idea could be a word, a group of words having a semantic meaning, an element of a drawing or an association of drawings and words. Those concepts were then tagged with codes, which were extracted from the data. As more data is collected, and re-reviewed, codes can be grouped into “items.” Common items were then grouped and defined under a new theme or sub-theme. Here, themes and sub-themes were not defined in advance.

Two socio-anthropologists (VR and JK) conducted a double analysis of each creation to produce items. Then, items were grouped into themes. Those analyses were conducted using a blind-assessment approach. The lists of themes were then compared and discussed by a group of experts consisting of: the two socio-anthropologists, an expert in education science (DB), a medical doctor and methodologist (VB), and a statistician (FT). This produced a single list of themes and sub-themes. The two analysts (VR and JK) then conducted a blind reclassification of all the items based on the new list. Their analyses were then compared. Any creation about which the two analysts disagreed on the attribution of items was examined by the multi-disciplinary group of experts so as to reach a consensus on the items and themes to be attributed.

### Statistical analysis

A descriptive analysis of the population of children concerned was established, followed by a descriptive analysis of each creation (frequency and percentage, median and inter-quartiles).

Univariate analyses were performed to compare the frequency of themes arising, according to the deprivation level of the child’s school. Multivariate analyses were performed using a Poisson regression model to identify the link between the number of themes per child and their deprivation status, adjusted for confounding factors (school class and gender). Statistical significance was denoted by an alpha of 0.05. All statistical analyses were done using the R system.

## Results

### Description

Data was collected from a total of 191 children from 8 classrooms in the 6 participating schools (Table 1). All of the children attending were aged between 9 and 12 and completed the study. Three of the 191 handed in a blank sheet. There were more boys than girls (101 vs 90). A majority of children were in fifth grade (106–55.5%), with the rest in sixth (61–31.9%) and fourth (24–12.6%) grades. With regard to social deprivation based on the EDI score, 42.4% of the children were classified as living in deprived areas and 57.6% of them were classified as living in non-deprived areas (Table 2).

**Table 1:** Description of the sample

School	Total no. school pupils	Rural/urban	European Deprivation Index	Deprived/non-deprived		REP +*	Classes taking part in the survey	No. of pupils taking part in the survey
1	218	Urban	3.77	Deprived		Yes	Fourth grade	24
2	228	Urban	0.88	Deprived		No	Fifth grade	22
3	176	Rural	− 1.92	Non-deprived		No	Fifth grade	24
4	233	Rural	− 1.33	Non-deprived		No	Fifth grade	60
5	844	Urban	− 0.304	Non-deprived		No	Sixth grade	26
6	226	Rural	1.66	deprived		Yes	Sixth grade	35

*The French Education Ministry ranks schools according to their “social index.” In schools ranked “REP,” 55% of pupils come from the least privileged socio-professional classes. In “REP+” ranked schools that percentage mounts to nearly 70% (the national average being around 40%)

**Table 2:** Initial socio-demographic characteristics

Child characteristics	*N* = 191	Frequency (%)
School class	Fourth grade	24 (12.6)
	Fifth grade	106 (55.5)
	Sixth grade	61 (31.9)
Gender	Girls	90 (47.1)
	Boys	101 (52.9)
Mean age (SD)		10.4 (0.8)
Geographic area	Rural	84 (44)
	Urban	107 (56)
Deprivation Level	Non-deprived	110 (57.6)
	Deprived	81 (42.4)

### Children’s creations (Table 3)

#### Good health

In response to the request to “write or draw anything you think keeps you healthy,” physical activity was found to be the most important factor for good health among almost all children (81.2%). The second biggest category was eating healthy food (71.2%): fruit and vegetables or greens accounting for 47.1% and also low sugar and dairy products, and low-salt and low-fat foods. Others were concerned with basic needs (51.3% of drawings): drinking (30.9%), sleeping (24.6%), controlling waste elimination (16.8%), accommodation (8.9%), eating (4.2%), breathing (3.7%) and clothing (2.6%). Hygiene was identified as being good for health in 23% of the drawings, not smoking in 15.7% and healthcare/vaccines in 13.6%. Other miscellaneous references to health factors were socio-psycho-affective: food, pleasure, and entertainment.

**Table 3:** Description of children’s drawings and writings. (Oakley’s categories for good and bad health are underlined)

Major theme		Sub-themes	Number of children quoting the item *N* (%) out of 191 (100%)
Good health			
Excercises and sports			*155 (81.2%)*
Diet-healthy food			*136 (71.2%)*
		Fruit and vegetables Water Low sugar Dairy products Low salt Low fat Balanced Diet	90 (47.1%) 32 (16.8%) 22 (11.5%) 16 (8.4%) 8 (4.2%) 6 (3.1%) 49 (25.7%)
Basic needs			*98 (51.3%)*
		Drink Sleep Poop and pee Have a home- nice home Eat Breathe Keeping warm-Clothes	59 (30.9%) 47 (24.6%) 32 (16.8%) 17 (8.9%) 8 (4.2%) 7 (3.7%) 5 (2.6%)
Hygiene			*44 (23%)*
Tobacco (not smoking)			*30 (15.7%)*
Healthcare/vaccines			*26 (13.6%)*
Psycho-socio-affective		Good mood	*21 (11%)*
Pleasure			*16 (8.4%)*
		Fun foods	*15(7.9%)*
Entertainment (TV, movies)			*(5.8%)*
Not being sick			*8 (4.2%)*
Other			*11 (5.8%)*
Mean (SD) number of themes identified per child; median			*3.9 (2); 4*
Bad health			
Diet			*94 (49.2%)*
		Too much sugar Fatty food Too salty Fruit and vegetables Palm oil Overweight–“being fat” Eating snacks	75 (39.3%) 30 (15.7%) 26 (13.6%) 7 (3.7%) 7 (3.7%) 29 (15.2%) 4 (2.1%)
Smoking			134 (70.2%)
Alcohol			114 (59.7%)
Sedentariness-lack of exercise			59 (30.9%)
Basic needs			*60 (31.5%)*
		Protection from cold–winter Not eating Not sleeping Not drinking enough	33 (17.3%) 15 (7.9%) 15 (7.9%) 5 (2.6%)
Disease			*37 (19.4%)*
Hygiene			*33 (17.3%)*
		Not washing yourself	29 (15.2%)
Taking drugs			*33 (17.3%)*
Environment/pollution			*24 (12.6%)*
Medicines			*22 (11.5%)*
Taking risks–sex without condoms			*20 (10.5)*
Seeking healthcare			*16 (8.4%)*
Emotional factors			*9 (4.7%)*
Death			*4 (2.1%)*
Mean (SD) number of items identified per child; median			*3.95 (2) 4*
Cancer			
Descriptions			*143 (74.9%)*
	By cancer sites		*117 (61.3%)*
		Lung Breast Heart Liver Hair Skin Everywhere Willy (penis) Brain Stomach Throat Blood other	73 (38.2%) 66 (34.6%) 38 (19.9%) 24 (12.6%) 14 (7.3%) 13 (6.8%) 12 (6.3%) 9 (4.7%) 8 (4.2%) 8 (4.2%) 6 (3.1%) 5 (2.6%) 23 (12%)
	By definition	A disease A serious disease	55 (28.8%) 32 (16.8%)
	By epidemiology	Age related disease Geographical location	*9 (4.7%)* *1 (0.5%)*
	By symptoms	(loss hair, heart failure, breathing problems)	*9 (4.7%)*
	By biological process	Tumor/cells	*6 (3.1%)*
	By level of knowledge	Do not know	*3 (1.6%)*
Risk factors			*89 (46.6%)*
	By behavioral causes		*86 (45%)*
		Tobacco Alcohol Food Drugs Others	73 (38.2%) 23 (12%) 12 (6.3%) 9 (4.7%) 5 (2.6%)
	By other causes		*11 (5.8%)*
		Sun Fate Others	4 (2.1%) 2 (1%) 5 (2.6%)
Outcomes			*73 (38.2%)*
		Always deadly Sometimes deadly	28 (14.7%) 45 (23.6%)
Consequences			*70 (36.6%)*
		Hair lost Sadness After-effects Pain Others	51 (26.7%) 20 (10.5%) 3 (1.6%) 3 (1.6%) 9 (4.8)
Cure/treatment			*54 (28.3%)*
		Hospital Curable disease Medics Surgery Doctor	30 (15.7%) 21 (11%) 17 (8.9%) 9 (4.7%) 3 (1.6%)
Prevention			*39 (20.4%)*
		Of smoking Of drinking alcohol Of other risks General guidelines	28 (14.7%) 11 (5.8%) 8 (4.2%) 8 (4.2%)
Personal history		Experience of friends or relatives having cancer	*4 (2.1%)*
Research			*4 (2.1%)*
Mean (SD) number of themes given per child; median			4.31 (2.2); 4

The values in italic emphasis refers to the major Themes

#### Bad health

In response to the request to “write or draw anything you think makes you unhealthy,” food featured in 49.2% of the children’s drawings with most of references to too much sugar, fat, and salt. There were again references to being overweight and snacking. The drawings also portrayed smoking, drinking alcohol (59.7%), sedentary lifestyles/lack of sport (30.9%), basic needs (31.5%), hygiene and drugs (31.5%), and pollution 12.6% as factors. Taking medication and seeking healthcare were also associated with bad health.

#### Cancer

**Fig. 1:**
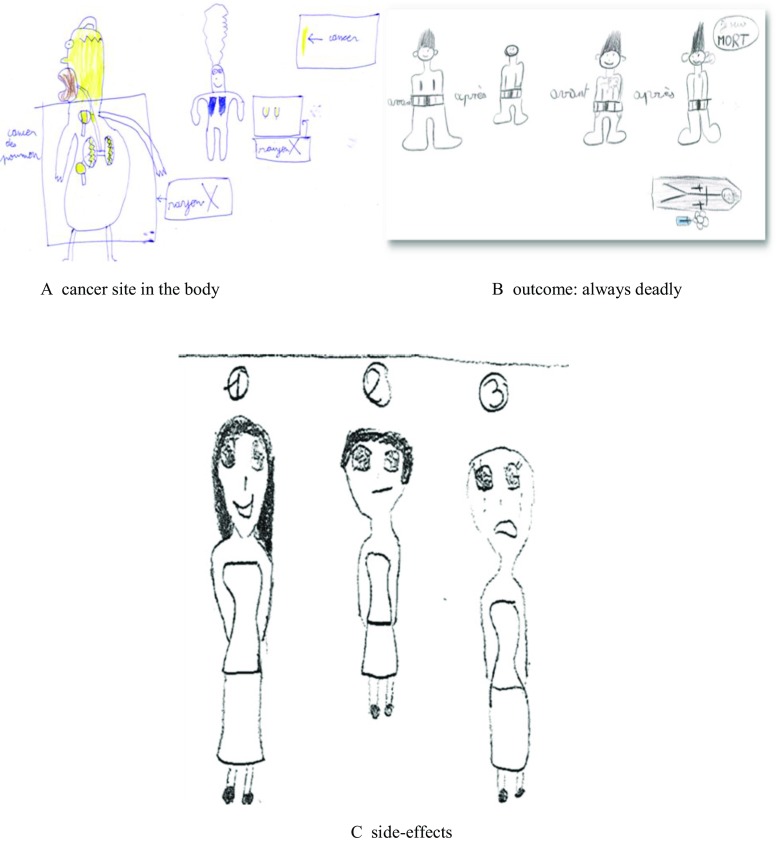
Examples of drawings about cancer. **a** Cancer site in the body. **b** Outcome, always deadly. **c** side-effects

In response to the request to “write or draw anything you know about cancer,” the analyses of the drawings revealed five major themes. The first theme associated with “cancer” is the description by “cancer site,” with 61.3% of children referring to the parts of the body in which cancer can develop. The affected organs were either represented anatomically or colored in/blackened out, and sometimes the drawings were of healthcare situations (Fig. 1a). Eighty-eight of the 117 children referring to cancer sites mentioned two or more sites. The most-frequently mentioned were lung cancer (38.2% of children), breast cancer (34.6%), heart cancer (19.9%), and liver cancer (12.2%). None of the children mentioned colorectal cancer. The notion of risk factors was the second most frequently addressed theme, and awareness among the children was high (46.6%), particularly in tobacco (38.2% of children), which was considered the main risk factor, followed by alcohol (12%), food (6.3%), and drugs (4.7%). The third most frequently addressed theme was “outcomes” (38.2%) among those who considered cancer to be deadly sometimes (23.6%) or always (14.7%), see Fig. 1b. Next, the consequences of the disease (36.6%) are associated with its side effects, for example, hair loss (26.7% of children) and sadness (10.5%) see Fig. 1c. The themes of “cure/treatment” and “prevention” were each respectively mentioned by 28.3% and 20.4% of the children. The issue of cancer prevention was addressed in message form, such as: “if you don’t want cancer don’t smoke,” and primarily focused on tobacco (14.7% of children).

The mean number of themes referenced per child was estimated at 4.31 (SD = 2.2).

### Comparison between deprived and non-deprived populations

The average number of items produced by the children according to their deprivation status is estimated at 3.6 (SD = 1.68) among children in deprived areas, and 4.83 (SD = 2.24) for non-deprived areas, with a significant statistical difference, *p* < 0.001 (Table 3). The number of items produced per child is also much higher among girls (4.78, SD = 2.13 vs 3.90, SD = 2.12; *p* = 0.004). The number of items increases the higher the school grade (4th grade—3.2, SD = 1.1 vs 5th grade—4.13, SD = 2.1 vs 6th grade—5.07, SD = 2.4; *p* = 0.0026).

After adjusting for gender, school class level, the link between the child’s deprivation status, and the mean number of themes addressed remains intact: non-deprived children express an estimated 0.37 items more than deprived children, which is a significant gap (*p* < 0.001) (see Table 4). We then conducted a comparative analysis of themes and sub-themes according to deprivation status (Table 5). There was a significant difference in the cancer sites identified according to a child’s deprivation status, with: lung cancer (47.3% non-deprived vs 25.9% deprived *p* = 0.004), heart cancer (28.2% non-deprived vs 8.6% deprived *p* = 0.0008), and liver cancer only being mentioned by non-deprived populations (12.5%; *p* < 0.0001). Non-deprived children more often list alcohol as a risk factor (17.3 vs 4.9%, *p* = 0.01). Deprived populations consider cancer to be “always deadly” more often than non-deprived children (27.2 vs 5.5% *p* < 0.0001). Non-deprived children were more likely to consider cancer to be curable than deprived children (18.2 vs 1.2%, *p* < 0.0001). Deprived children identified more themes with a high stigmatizing meaning such as the theme “always deadly” or themes referring to side effects.

**Table 4:** Association between deprivation status, other confounding factors and number of themes identified per child. Univariate (Wilcoxon test) and multivariate analysis (Poisson regression)

Variables tested	Univariate analysis		Multivariate analysis		
	Themes identified, mean (SD)	*p* value	Point estimate	*p* value	
Deprivation status					
Deprived	3.6 (1.68)		ref.		
Non-deprived	4.83 (2.24)	< 0.001	0.37	< 0.001	
Gender					
Girls	4.78 (2.13)		ref.		
Boys	3.9 (2.12)	0.004	− 0.15	0.04	
School class					
6	5.07 (2.4)		ref		< 0.001
5	4.13 (2.1)		− 0.24	0.08	
4	3.2 (1.1)	0.003	− 0.32	< 0.001	
Geographical area			–	–	
Rural	4.53 (2.0)				
Urban	4.14 (2.3)	0.09

**Table 5:** Association between deprivation status and frequency of themes identified. Univariate analysis (Wilcoxon test), significant results only. PR translates the number of themes identified by deprived children, compared to the non-deprived. For example, for the lung cancer, deprived children identify half less items than non-deprived children

Themes identified	Whole population *N* (%)	Non-deprived population *N* (%)	Deprived population *N* (%)	Crude PR [IC95%]	*p* value
Cancer site: lung	73 (38.2)	52 (47.3)	21 (25.9)	0.55 [0.37–0.81]	0.004
Cancer site: heart	38 (19.8)	31 (28.2)	7 (8.6)	0.31 [0.15–0.61]	0.0008
Cancer site: liver	24 (12.5)	24 (12.5)	0 (0)		< 0.0001
A disease	55 (28.7)	43 (39.1)	12 (14.8)	0.38 [0.23–0.64]	0.0003
A serious disease	32 (16.7)	27 (24.5)	5 (6.2)	0.25 [0.11–0.56]	0.0007
Behavioral causes: alcohol	23 (12)	19 (17.3)	4 (4.9)	0.29 [0.11–0.74]	0.01
Outcomes: always deadly	28 (14.6)	6 (5.5)	22 (27.2)	4.98 [2.35–10.55]	< 0.0001
Outcomes: sometimes deadly	45 (23.5)	41 (37.3)	4 (4.9)	0.13 [0.06–0.28]	< 0.0001
Visible side-effects	63 (32.9)	28 (25.5)	35 (43.2)	1.7 [1.14–2.54]	0.01
Visible side-effects: hair loss	51 (26.7)	19 (17.3)	32 (39.5)	2.29 [1.43–3.67]	0.0008
Invisible side-effects	69 (36.1)	31 (28.2)	38 (46.7)	1.66 [1.14–2.42]	0.0095
Cure/treatment: a curable disease	21 (10.9)	20 (18.2)	1 (1.2)	0.07 [0.02–0.28]	< 0.0001

*PR* prevalence ratio

## Discussion

This is the first study to involve such a large cohort of 9–12 year olds and to identify significant differences in representations of cancer between deprived and non-deprived children. From the age of nine, deprived children have radically different views about the key representations of cancer: they are more likely to believe the illness is systematically deadly (27.2 vs. 5.5%, *p* < 0.0001). They are less likely to believe it is a treatable illness (1.2 vs 18.2%, *p* < 0.0001). They are less likely to associate cancer with risky behaviors, particularly alcohol consumption. All of which reflects a fatalistic outlook. Non-deprived children are able to generate more ideas and content on the subject of cancer than deprived children (number of items mentioned: 4.8 vs 3.6 *p* < 0.001). This difference also persists independently of other factors that could potentially influence a child’s capacity for self-expression. The wider the variety of items comprising the representation, the more easily the latter is connected to other items of knowledge [6]. Inequalities can exacerbated by the fact that deprived children may live in environments leaving them more exposed to risks and tend to be less able to integrate information about prevention.

### Other lessons from this study

An examination of the main representations of cancer featured in the children’s drawings/writings reveals that the disease is primarily seen as an illness of the organs, implying that they have a segmented, anatomo-clinical perception of cancer. This representation may conflict with the global approach to the body and health that is widely being promoted in programs as the cornerstone of the government’s National Health and Nutrition plan. Indeed, this global view is visible in the children’s drawings of good and bad health, which show physical and eating behaviors consistent with the catchphrases associated with those programs. Strictly healthcare-related items, such as doctors, hospitals, and vaccines, were under-represented in the drawings. Another strand of data confirms this observation: although physical activity is identified by children as the leading factor for good health and is seen as influencing bad health in 30% of the children, the concept of sedentariness is completely absent from the cancer risk factors identified by the children. They therefore seem to struggle to connect the idea of general prevention in healthcare with that of an illness described as attacking a specific organ. Representing cancer more as an illness of blood cells than of an organ, or as an illness linked with general aging in the body would be more consistent with the representative base children already have with regard to health.

As regards knowledge of risk factors, our research therefore leads us to consider that most of the population is health literate when it comes to tobacco-related risks. Indeed, this knowledge was identified in younger children (4–8 year olds) in the Porcellato study [19]. However, alcohol consumption is spontaneously identified as a risk factor among 38% of non-deprived children, but in just 12% of deprived children, and the trend is similar when it comes to food (21.6 vs. 6.1%). Accordingly, non-deprived children present richer representations of risk factors that are more consistent with scientific data than those of deprived children. Specifically, the association between alcohol and liver cancer has only been reported by non-deprived children. This may be connected to representations of female alcoholism, which tends to affect women with higher levels of education. More importantly, however, representations of these women are predominated by the idea of personal consequences. [3].

Finally, with regard to the illness itself, which was a highly visible theme in the children’s creations, the differences were striking and clearly suggest that children in deprived areas equate cancer with death. This deterministic view is consistent with secondary prevention avoidance behaviors, as seen in publications [14]. It is therefore understandably difficult for such populations to participate in cancer screening programs when the announcement of an “anomaly” would be translated as one of imminent and inevitable death.

This study provides statistical validation of the data suggested in the research conducted by Oakley [17] and Knighting [9] using the same methodology and addressing the same age bracket. With respect to representations of cancer, we found the same key themes (cancer site, risks, outcomes, and consequences) ranked in the same order of appearance. The sample used in the Knighting study included social differences based on the Carstairs and Morris Index of Deprivation. While we reproduced the structure of this sample, applying the European deprivation index, unlike Knighting, we were able to demonstrate statistically that social inequalities, in terms of representations and health literacy, are present from childhood. These factors stand to play an important role in maintaining social inequality over the years to come, when those children become adults and will need to make decisions about proposed primary and secondary prevention measures. Several other conclusions reached in our study will need to be taken into consideration in drawing up and implementing education programs in schools.

Physical activity is now widely recognized as a prevention practice that should be promoted for every human being. The WHO developed the “Global Recommendations on Physical Activity for Health” [8] in order to provide, for all the age groups, guidance on the type and amount of physical activity needed for the prevention of non-communicable diseases. Although those guidelines are widely disseminated in mass media and recognized by all, they remain less frequently implemented according to a socio-economic gradient. This has been observed in a specific child population type: those living in the most deprived circumstances are less likely to undergo physical activity than those living in the least deprived [13].

Promotion of physical activity should be performed adequately, through intervention aiming at addressing targeted population issues and preferably in the workplace. That intervention is even more likely to tackle inequalities when implemented at a high social or policy decision-making level [11]. For children, such results highlight the need to develop educational efforts, endorsed by the national school system, performed in the school environment, aiming at promoting healthy behaviors such as physical activity and fighting negative health representations, such as the image of cancer as being systematically deadly. Indeed, studies of adults have found the representation of cancer as a deadly disease among the more deprived classes [22]. Our study demonstrates that the representation exists even before adolescence, which means that early intervention is to be favored.

Our study was limited by a number of factors. One of these stems from the method chosen, i.e. “write and draw,” which involved interpreting and ranking the drawings by theme. Had we not been limited by the time available for collecting data, we could have added the “Draw, write and tell” system, as described by Angell [1]. That said the bias created in ranking the themes was offset by having several readers validate the themes. Similarly, the deprived/non-deprived classification method used is based on environmental indicators. Individual indicators, such as the socio-professional category of the children’s parents, would have yielded more reliable results, but require additional authorizations and, a high level of quality control of the data collected. Another limit of this work is that the ethnicity and immigration status of the children could not have been collected, this being forbidden under French law. Those variables could have helped to more precisely identify differences in children’s representations.

## Conclusion

Our research shows that most children have good knowledge and appropriate representations of health, cancer, and risk factors. Tobacco particularly, is adequately identified as the most important and avoidable risk factor. More importantly, however, differences highlighted between child deprivation levels alert us in showing that social inequalities affect those representations as early as childhood.

This study strongly advocates the development of early cancer prevention programs in schools and particularly targeted programs in schools in deprived areas, to avoid inequalities between deprived and non-deprived children becoming ingrained. It encourages decision-makers to develop and implement specific educational strategies that take better account of individual child perceptions in order to foster healthy behavior, especially in relation to cancer. Researchers are legitimate in providing their expertise in educational and behavioral sciences in order to help those intervention efforts fulfill this goal.
